# Early High‐Frequency Network Reorganization After Spinal Cord Stimulation Is Linked to Long‐Term Recovery in Disorders of Consciousness

**DOI:** 10.1002/cns.71101

**Published:** 2026-08-22

**Authors:** Kang Zhang, Yunqian Li, Zean Li, Xinran Zhang, Zhiyang Xu, Chunyun Zhang, Chuanxiang Lv, Boyuan Zhang, Haoyong Jin, Li Bie

**Affiliations:** ^1^ Department of Neurosurgery The First Hospital of Jilin University Changchun China; ^2^ Department of Neurosurgery The Fourth People's Hospital of Jinan Jinan China; ^3^ Department of Neurosurgery Qilu Hospital of Shandong University (Qingdao) Qingdao China; ^4^ Department of Neurosurgery The First Affiliated Hospital of Guangzhou Medical University Guangzhou China

**Keywords:** alpha/beta oscillations, brain‐network reorganization, candidate recovery markers, disorders of consciousness, ModularAUC, NetPower, source‐space EEG, spinal cord stimulation

## Abstract

**Aims:**

Spinal cord stimulation (SCS) is a promising treatment for disorders of consciousness (DoC), but early neurophysiological markers of durable recovery remain unclear. We aimed to determine whether source‐space EEG network energy and communication features capture early brain‐network reorganization linked to 6‐month recovery in chronic DoC.

**Methods:**

Thirty‐one chronic DoC patients undergoing cervical SCS completed resting‐state EEG before stimulation and at 1 week and 1 month after stimulation onset. Source‐space EEG was analyzed across delta, theta, alpha, and beta bands. NetPower quantified network energy within five canonical networks, and ModularAUC quantified threshold‐robust within‐ and between‐network communication. EEG features were related to 6‐month CRS‐R outcomes.

**Results:**

Recovery was associated with sustained alpha/beta NetPower enhancement across default mode, frontoparietal, cingulo‐opercular, somatomotor, and occipital networks, peaking at 1 month. Beta‐band ModularAUC showed the clearest communication‐architecture consolidation, with broader within‐ and cross‐network synergy in recovered patients. Low‐frequency changes were weaker and less consistent. High‐frequency NetPower features, especially FPN alpha at 1 month and CON beta at 1 week, showed the strongest associations with long‐term improvement.

**Conclusions:**

SCS‐related recovery in DoC is characterized by high‐frequency network‐energy enhancement and beta‐band communication consolidation. Here, high‐frequency network reorganization mainly refers to alpha‐ and beta‐band source‐space EEG changes. Source‐space EEG may provide early potential neurophysiological markers for monitoring recovery.

## Graphical abstract

Source‐space EEG revealed that recovery after spinal cord stimulation in chronic disorders of consciousness is marked by early consolidation of high‐frequency network activity. Sustained alpha/beta NetPower increases and beta‐band communication strengthening across large‐scale networks—strongest at 1 month—were associated with better 6‐month clinical recovery.

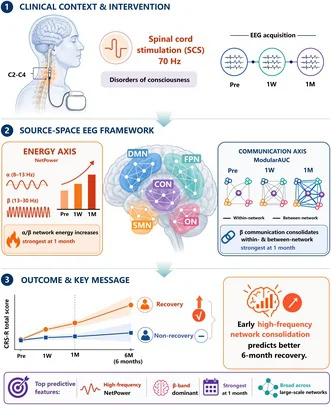

## 1 Introduction

Disorders of consciousness (DoC), including vegetative state/unresponsive wakefulness syndrome (VS/UWS) and minimally conscious state (MCS), remain among the most challenging syndromes after acquired brain injury [1, 2, 3]. MCS can be further divided into MCS− and MCS+ according to language‐related responses such as command following [4, 5]. Clinical decisions still rely heavily on the Coma Recovery Scale‐Revised (CRS‐R), which is indispensable but inherently behavior dependent [6, 7]. In these patients, arousal fluctuation, motor‐output impairment, aphasia, fatigue, and limited behavioral repertoire can mask residual brain function [1]. As a result, short‐term clinical examination may underestimate recovery potential, and delayed improvement may be recognized only after a substantial period of treatment and observation.

Neuromodulation has become a major therapeutic direction for DoC because it aims to reactivate residual circuits rather than merely describe the behavioral state [8]. Non‐invasive methods such as transcranial direct current stimulation (tDCS) or transcranial alternating current stimulation (tACS) can modulate cortical excitability, whereas invasive approaches may engage deeper arousal circuits [9, 10]. Among invasive approaches, spinal cord stimulation (SCS) has emerged as a representative arousal‐promoting strategy with demonstrated potential to improve the level of consciousness. A large‐sample study reported that 54% of VS patients achieved favorable outcomes after long‐term SCS [11]; a subsequent 2019 study found that 42% of VS patients were SCS‐responsive [12]. Synthesizing recent reports, the overall SCS arousal‐response rate in DoC is approximately 30%–50%. Thus, although SCS holds promise, its therapeutic effect is markedly heterogeneous, and early behavioral changes are often difficult to distinguish from transient arousal fluctuations. Mechanistically, it is proposed that SCS promotes arousal by engaging ascending arousal systems, thalamocortical modulatory pathways, and large‐scale brain‐network activity [13, 14, 15]. Resting‐state EEG evidence from Bai et al. suggests that SCS may modulate a “reticular formation‐thalamus‐frontal cortex” network in MCS patients [16]; Yamamoto et al. reported that SCS broadly increases whole‐brain cerebral blood flow, with a 22% elevation during stimulation relative to pre‐stimulation [17]. Consequently, the pivotal question facing the field is no longer simply whether SCS is effective, but rather whether it induces central biomarkers that are linked to long‐term recovery and whether these biomarkers can be objectively captured before substantial behavioral improvement manifests.

Electroencephalography (EEG) and functional magnetic resonance imaging (fMRI) have been widely used to support clinical assessment and mechanistic investigation in DoC. Whole‐brain network models derived from resting‐state fMRI have provided a vital platform for understanding brain functional plasticity [18, 19, 20, 21]; graph‐theoretic analyses can characterize topological properties of functional networks, including local and global communication efficiency, while modularity analysis reveals organizing principles of within‐ and between‐network connectivity [22, 23]. Relative to fMRI, EEG offers bedside accessibility, repeatability, and millisecond‐scale temporal resolution [24], making it especially suited to capturing rapidly evolving system‐level brain dynamics following neuromodulation. Prior studies show that patients with higher levels of consciousness or better outcomes tend to exhibit more preserved connectivity and better organized network dynamics [25, 26]. At the same time, neuromodulation studies suggest that therapeutic response reflects not only regional activation changes but also reconfiguration of cross‐regional and cross‐network information integration [27, 28, 29, 30]. Yet most EEG studies remain centered on scalp‐level spectral power, single connectivity metrics, or isolated dynamic features [31, 32, 33], limiting their ability to capture both network activation and network communication.

To address this gap, we developed a source‐space EEG dual‐axis framework to capture early brain‐network reorganization after SCS. This framework quantified network energy using spectral power within five canonical networks and communication architecture using threshold‐robust module‐interaction AUC. We then related longitudinal EEG changes from baseline to 1 week and 1 month after implantation to 6‐month clinical recovery.

## 2 Methods

### 2.1 Study Design and Participants

This longitudinal observational study included 31 patients with chronic DoC who underwent cervical SCS as clinical treatment and completed resting‐state EEG at all three predefined time points plus 6‐month follow‐up. Inclusion criteria were DoC diagnosed by repeated CRS‐R assessment, age 18–75 years, disease duration of at least 3 months, clinical stability with limited response to conventional treatment, and no major skull defect affecting EEG acquisition. Exclusion criteria included disease duration under 3 months, vertebral fracture or severe spinal canal stenosis, severe structural defects compromising assessment, intracranial implants, life‐threatening complications, or anesthetic/sedative use within 48 h before EEG.

### 2.2 SCS Implantation and Stimulation Protocol

SCS electrodes were implanted under general anesthesia into the dorsal epidural space at the C2–C4 level. Postoperative imaging and clinical examination were used to confirm electrode location and treatment safety. Stimulation was initiated on postoperative day 3 and delivered daily from 08:00 to 20:00 using alternating 15‐min ON and 15‐min OFF cycles. Stimulation parameters were set at 70 Hz, a pulse width of 210 μs, and 1.2–5.4 V, and were individually adjusted according to patient tolerance and arousal‐related responses while avoiding excessive motor activation. EEG was acquired during quiet resting state at baseline/pre‐SCS (Pre), 1 week after stimulation initiation (Post1W), and 1 month after stimulation initiation (Post1M). The stimulator was turned off 15 min before each recording to minimize online stimulation artifacts.

### 2.3 Clinical Assessment and Outcome Definition

CRS‐R was used for diagnosis and longitudinal outcome assessment. Baseline variables included age, sex, etiology, disease duration, CRS‐R total score, and CRS‐R index [34]. The primary categorical outcome was defined as improvement by at least one CRS‐R diagnostic category at 6 months relative to baseline. Patients meeting this criterion were assigned to the Recovery group; the remainder formed the Non‐recovery group. Two continuous endpoints quantified improvement magnitude: ΔCRS‐R total at (6 m − baseline) (defined as CRS‐R total at 6 months minus CRS‐R total at baseline) and Relative ΔCRS‐R index (defined as 100 × [CRS‐R index at 6 months − CRS‐R index at baseline]/CRS‐R index at baseline). The former captures the absolute magnitude of behavioral improvement; the latter reflects the relative recovery efficiency.

### 2.4 EEG Acquisition and Preprocessing

EEG was recorded with a 64‐channel Grael system (Compumedics, Australia) at 1024 Hz using the international 10–20 layout. Each session lasted approximately 15 min during quiet rest, with impedance maintained below 10 kΩ. Preprocessing was performed in MATLAB R2022b with EEGLAB [35] and custom scripts. Bad channels were identified by visual inspection and signal‐quality criteria, including flat signals, high noise, disconnection, or abnormal amplitude/variance relative to neighboring electrodes, and were removed before ICA. Data were band‐pass filtered at 1–40 Hz and notch filtered at 48–52 Hz. ICA components corresponding to ocular, muscle, cardiac, and electrode artifacts were removed by integrating topography, time course, and spectrum. Only artifact‐free segments within −100 to 100 μV were retained, and each included recording preserved more than 80% of its original duration.

### 2.5 Source Reconstruction and Network Definition

Source‐space analysis employed a fixed 6‐s time window to obtain stable individual‐level network phenotypes. Source reconstruction was performed using the FieldTrip toolbox [36] with a partial canonical coherence (PCC) frequency‐domain beamformer. The standard boundary element method (BEM) head model was adopted, with a source‐space grid resolution of 0.6 cm and a regularization parameter of 10%. Noise projection was enabled, source orientation was fixed, and the spatial filter was retained for subsequent ROI‐level aggregation. Four frequency bands were defined: delta (1–4 Hz), theta (4–8 Hz), alpha (8–13 Hz), and beta (13–30 Hz). ROIs were defined according to the Dosenbach atlas [37] and mapped onto five canonical functional networks: default mode network (DMN), frontoparietal network (FPN), cingulo‐opercular/salience network (CON), somatomotor network (SMN), and occipital network (ON). A schematic of the brain template used for source reconstruction and network mapping is provided in Figure S1.

### 2.6 Construction of the Dual‐Axis EEG Phenotype

Two complementary source‐space EEG phenotypic axes were constructed. All source‐space features were first estimated from retained clean 6‐s EEG segments and then averaged within each subject to generate subject‐level phenotypes. The first is the energy‐state axis. For each frequency band, frequency‐domain source estimates were obtained using a multitaper Fourier transform with discrete prolate spheroidal sequence (DPSS) tapers, and source power was extracted as the largest eigenvalue of the source power matrix, as implemented in FieldTrip [36]. Spectral power was first computed for each ROI and then averaged within each of the five canonical networks, yielding network‐level power indices. To mitigate the influence of zero values and skewed distributions, a small constant was added before log transformation. The resulting network‐level energy metric is denoted NetPower. The second is the communication‐architecture axis. For this axis, source‐space functional connectivity matrices were constructed from segment‐level source Fourier coefficients using coherence, and the absolute value of the imaginary component of coherence was retained to reduce the potential influence of zero‐lag coupling and volume conduction. Based on the source‐space functional connectivity matrix, 15 module‐interaction features were extracted per frequency band, comprising 5 within‐network and 10 between‐network connectivity indices. To reduce the bias associated with any single graph‐threshold selection, module‐interaction strength was computed using GRETNA [38] across 55 thresholds ranging from 0.06 to 0.60. The area under the curve was calculated by integrating the threshold‐dependent module‐interaction values across this threshold range and was taken as the final threshold‐robust phenotype, denoted ModularAUC. The same analytical pipeline was applied to all four frequency bands.

### 2.7 Statistical Analysis and Correlation Analysis

For demographic comparisons, sex and etiology were tested using *χ* ^2^ tests, whereas age, post‐injury duration, CRS‐R total score, and CRS‐R index were compared using *t*‐tests. Data normality was assessed with the Shapiro–Wilk test. For the energy axis, linear mixed‐effects models tested the effects of Group, Time, and Group × Time, with subject as a random intercept. NetPower differences between Recovery and Non‐recovery groups were then assessed for each network, time point, and frequency band, with Cohen's d used as the effect size. For the communication axis, ModularAUC was compared between groups at three static time points (Pre, Post1W, Post1M) and three change windows (ΔPost1W − Pre, ΔPost1M − Pre, ΔPost1M − Post1W) using two‐tailed Welch's *t*‐tests, with Hedges' *g* as the effect size. Benjamini–Hochberg false discovery rate correction was applied within prespecified analytical families. For NetPower group comparisons, each frequency band was treated as a separate test family, and post hoc between‐group comparisons were corrected across the five networks and three time points within that band. For ModularAUC group comparisons, correction was performed separately within each frequency band and comparison type, with the 15 module‐interaction features corrected within static time‐point comparisons and change‐window comparisons. For EEG–clinical correlation analyses, correction was performed separately for each clinical endpoint and feature family, namely NetPower‐alpha, NetPower‐beta, ModularAUC‐alpha, and ModularAUC‐beta.

Associations between EEG features and long‐term clinical improvement were examined using partial Spearman rank correlations controlling for age, time post‐injury, and baseline CRS‐R total score. Analyses included alpha‐ and beta‐band NetPower and ModularAUC features and two continuous clinical outcomes: ΔCRS‐R total and Relative ΔCRS‐R index. Clinical, NetPower, and ModularAUC data were aligned by subject to form a unified analysis matrix. For each endpoint, the top 10 EEG features ranked by absolute correlation coefficient were identified and tested for robustness using 5000‐sample bootstrap confidence intervals, leave‐one‐out stability analysis, and feature overlap/composition analysis across feature family, time point, and feature type. The same covariate adjustment was retained in all bootstrap and leave‐one‐out analyses. All analyses were performed in Python.

## 3 Results

### 3.1 Demographic and Clinical Characteristics

**FIGURE 1:**
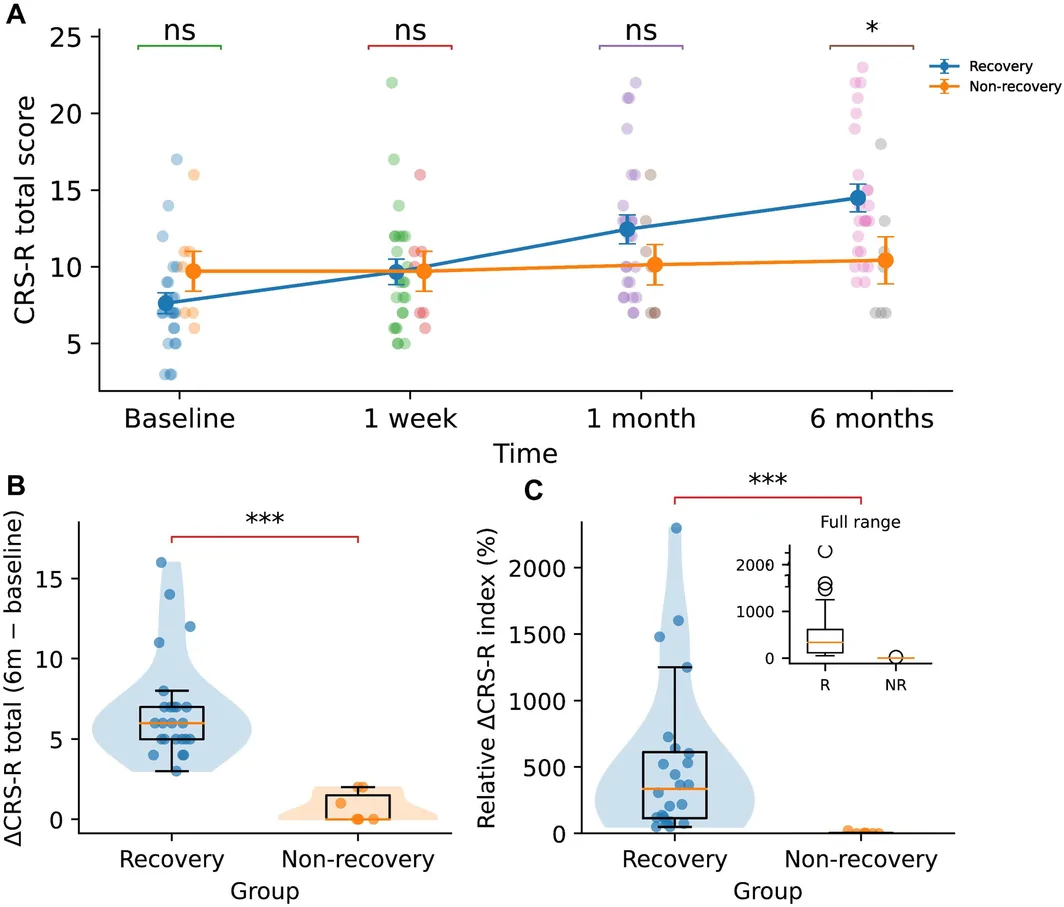
Baseline comparability and long‐term clinical outcome differences between the Recovery and Non‐recovery groups. (A) Longitudinal CRS‐R total scores from baseline to 6 months show progressive behavioral improvement in the Recovery group but limited change in the Non‐recovery group. (B) Absolute behavioral improvement, measured as ΔCRS‐R total (6 m − baseline), is greater in the Recovery group. (C) Relative behavioral improvement, measured as Relative ΔCRS‐R index (%), is also greater in the Recovery group. CRS‐R, Coma Recovery Scale–Revised. Δ indicates change between two time points. Recovery indicates patients whose 6‐month CRS‐R diagnostic category improved by at least one level relative to baseline, whereas Non‐recovery indicates patients without such improvement. ns indicates not significant. *p < 0.05, ***p < 0.001.

Baseline characteristics were comparable between the Recovery and Non‐recovery groups, with no significant differences in age (52.17 ± 13.77 vs. 50.86 ± 14.87 years, *p* = 0.62), female proportion (41.7% vs. 42.9%, *p* = 1.00), disease duration (19.83 ± 7.92 vs. 16.43 ± 7.16 months, *p* = 0.46), baseline CRS‐R total score (7.62 ± 3.33 vs. 9.71 ± 3.45, *p* = 0.16), baseline CRS‐R index (18.86 ± 17.79 vs. 28.52 ± 26.09, *p* = 0.29), or etiology distribution (*p* = 0.10) (Table 1). Early behavioral trajectories were largely overlapping, with no significant group difference in CRS‐R total score from baseline to 1 month; however, by 6 months, CRS‐R total was significantly higher in the Recovery group (14.50 ± 4.39 vs. 10.43 ± 4.08, *p* = 0.03) (Figure 1A; Table 1). Improvement indices showed clearer separation: the Recovery group had greater ΔCRS‐R total (6.88 ± 3.25 vs. 0.71 ± 0.95, *p* < 0.001) (Figure 1B; Table 1) and Relative ΔCRS‐R index (515.38 ± 582.08% vs. 3.97 ± 7.90%, *p* < 0.001) (Figure 1C; Table 1), as well as higher 6‐month CRS‐R index (57.28 ± 26.91 vs. 29.16 ± 26.58, *p* = 0.03) and ΔCRS‐R index (38.43 ± 20.67 vs. 0.64 ± 0.86, *p* < 0.001) (Table 1).

**TABLE 1:** Demographic and CRS‐R characteristics.

	Recovery (*N* = 24)	Non‐recovery (*N* = 7)	*p*
**Age (years)**			0.62
Mean (SD)	52.17 (13.77)	50.86 (14.87)	
Median [min, max]	55.00 [16.00, 68.00]	50.00 [33.00, 73.00]	
**Sex**			1.00
F	10 (41.7%)	3 (42.9%)	
M	14 (58.3%)	4 (57.1%)	
**Etiology**			0.10
Stroke	8 (33.3%)	4 (57.1%)	
TBI	14 (58.3%)	1 (14.3%)	
Hypoxia	2 (8.3%)	2 (28.6%)	
**Time post‐injury (months)**			0.46
Mean (SD)	19.83 (7.92)	16.43 (7.16)	
Median [min, max]	20.50 [9.00, 36.00]	14.00 [9.00, 27.00]	
**CRS‐R total score (mean (SD))**			
CRS‐R_total at baseline_	7.62 (3.33)	9.71 (3.45)	0.16
CRS‐R_total at 6m_	14.50 (4.39)	10.43 (4.08)	0.03
ΔCRS‐R_total at (6m−baseline)_	6.88 (3.25)	0.71 (0.95)	< 0.001
**CRS‐R index (mean (SD))**			
CRS‐R_index at pre_	18.86 (17.79)	28.52 (26.09)	0.29
CRS‐R_index at 6m_	57.28 (26.91)	29.16 (26.58)	0.03
ΔCRS‐R_index at (6m−pre)_	38.43 (20.67)	0.64 (0.86)	< 0.001
Relative ΔCRS‐R index (%)^a^	515.38 (582.08)	3.97 (7.90)	< 0.001

*Note:* Values are mean (SD) unless otherwise indicated.

Abbreviations: CRS‐R, Coma Recovery Scale–Revised; TBI, traumatic brain injury.

^a^ Relative ΔCRS‐R index (%) = 100 × (CRS‐R index at 6 months − CRS‐R index at baseline)/CRS‐R index at baseline.

### 3.2 NetPower: Recovery‐Related High‐Frequency Energy Enhancement Peaks at 1 Month

**FIGURE 2:**
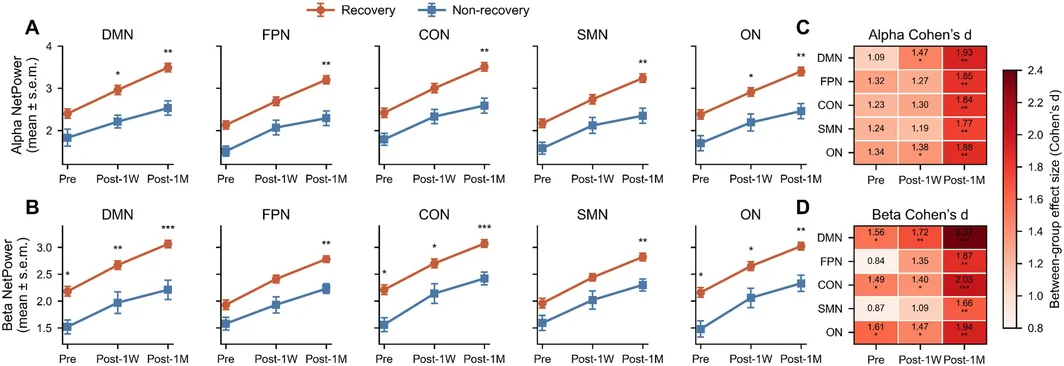
Recovery‐related enhancement of high‐frequency network energy after spinal cord stimulation. (A) Alpha‐band NetPower increases over time and remains higher in the Recovery group across the five canonical networks. (B) Beta‐band NetPower shows a similar but stronger pattern of increase and between‐group separation. (C) Effect‐size summary of alpha‐band NetPower indicates that between‐group separation is most pronounced at Post1M. (D) Effect‐size summary of beta‐band NetPower shows stronger and more widespread between‐group differences, especially at Post1M. NetPower denotes network‐level spectral power after log transformation. CON, cingulo‐opercular network; DMN, default mode network; FPN, frontoparietal network; ON, occipital network; Post1M, 1 month after stimulation onset; Post1W, 1 week after stimulation onset; Pre, pre‐implantation baseline; s.e.m., standard error of the mean; SMN, somatomotor network. Cohen's *d* indicates between‐group effect size (Recovery − Non‐recovery). *p < 0.05, **p < 0.01, ***p < 0.001. Stars indicate significance after Benjamini–Hochberg false discovery rate correction.

Recovery‐related separation was concentrated in the alpha and beta bands and was most evident at Post1M. In the alpha band, NetPower increased over time across all five canonical networks and remained consistently higher in the Recovery group than in the Non‐recovery group, indicating a pan‐network recovery‐related high‐frequency energy advantage (Figure 2A). Although positive group separation was already present at Pre and Post1W, it peaked at Post1M. The effect‐size heatmap confirmed large Post1M effects across DMN, FPN, CON, SMN, and ON, with Cohen's d of approximately 1.93, 1.85, 1.84, 1.77, and 1.88, respectively, all indicating widespread alpha‐band separation at 1 month (Figure 2C).

The beta band showed a similar but stronger pattern. NetPower was higher in the Recovery group across all networks and time points, with the most pronounced separation at Post1M, particularly in DMN, CON, and ON (Figure 2B). Post1M effect sizes were larger than those in the alpha band, reaching approximately 2.37 in DMN, 2.03 in CON, and 1.94 in ON; FPN and SMN also showed large effects of approximately 1.87 and 1.66, respectively (Figure 2D). All reported significant NetPower differences were corrected for multiple comparisons using the Benjamini–Hochberg false discovery rate procedure. Thus, beta‐band NetPower provided the strongest energy‐axis discrimination between outcome groups, with a cross‐network pattern that progressively consolidated by 1 month.

By contrast, theta and delta bands showed weaker and qualitatively different profiles. NetPower generally decreased over time in both groups, while the Non‐recovery group often retained higher absolute low‐frequency power, leading to predominantly negative effect sizes (Figure S2A–D). In the theta band, decreases from Pre to Post1M were observed across DMN, FPN, CON, SMN, and ON, with relatively larger group differences at Post1W; however, most effects were mild to moderate and did not survive FDR correction (Figure S2A,C). Delta‐band results were similar, with more evident negative separation at Post1W in DMN, FPN, SMN, and ON, but these effects were not stable after correction and lacked the cross‐network consistency and progressive strengthening observed in the alpha/beta bands (Figure S2B,D).

### 3.3 Communication Architecture: Beta‐Band Within‐Network Cohesion and Cross‐Network Synergy as the Core Recovery‐Related Pattern

**FIGURE 3:**
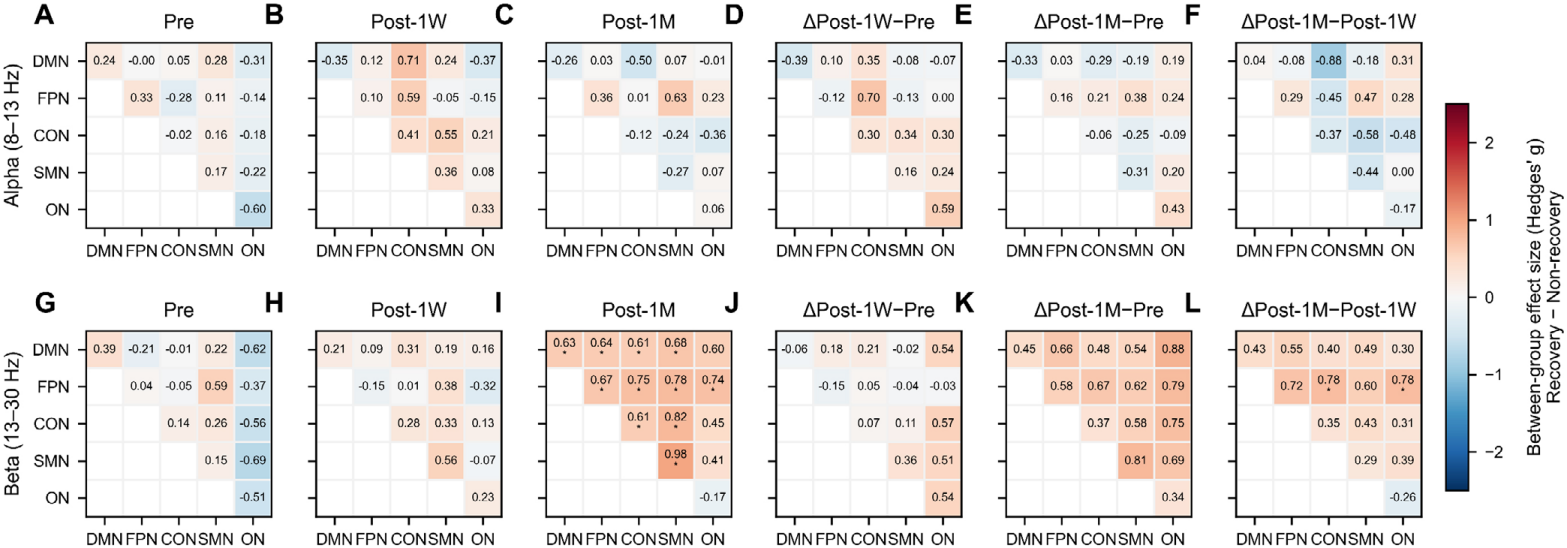
Recovery‐related reorganization of communication architecture in the alpha and beta bands. (A–F) Alpha‐band module‐interaction AUC shows limited and spatially heterogeneous between‐group differences across static time points and change windows. (G–L) Beta‐band module‐interaction AUC shows broader and more consistent recovery‐related enhancement, with the strongest between‐group separation at Post1M and in the later change windows. AUC, area under the threshold curve; CON, cingulo‐opercular network; DMN, default mode network; FPN, frontoparietal network; ON, occipital network; Post1M, 1 month after stimulation onset; Post1W, 1 week after stimulation onset; Pre, pre‐implantation baseline; SMN, somatomotor network. Δ indicates change between two time points. Hedges' *g* indicates between‐group effect size (Recovery − Non‐recovery). *p < 0.05 . Stars indicate significance after Benjamini–Hochberg false discovery rate correction.

Alpha‐band ModularAUC showed limited and heterogeneous communication‐architecture differences. At Pre, only sparse connections showed mild positive or negative effects, with no clear group separation (Figure 3A). At Post1W, moderate effects appeared in selected cross‐network edges, including DMN–CON, FPN–CON, and CON–SMN, suggesting early but restricted communication enhancement in the Recovery group (Figure 3B). By Post1M, alpha‐band effects remained inconsistent: FPN–SMN and FPN–ON showed positive separation, whereas DMN–CON and CON–ON shifted negatively, indicating edge‐specific rather than globally organized reconfiguration (Figure 3C). Change‐window analyses showed similarly scattered positive effects in ΔPost1W − Pre and ΔPost1M − Pre, with partial reversal in ΔPost1M − Post1W, supporting limited temporal stability of alpha‐band communication changes (Figure 3D–F).

Among the communication‐architecture features, beta‐band ModularAUC showed the most consistent exploratory recovery‐related pattern. Static effects were modest at Pre and Post1W (Figure 3G,H), but became broadly positive at Post1M across within‐ and between‐network connections involving DMN, FPN, SMN, and ON (Figure 3I). Positive effects included DMN–DMN, DMN–FPN, DMN–CON, DMN–SMN, DMN–ON, FPN–FPN, FPN–CON, FPN–SMN, and FPN–ON; notably, SMN–SMN approached Hedges' *g* ≈1.0, while FPN–SMN, FPN–ON, and CON–SMN reached moderate‐to‐strong effects (Figure 3I). Change‐window analyses indicated limited early effects in ΔPost1W − Pre, mainly in selected cross‐network and ON‐related edges (Figure 3J), but broader positive changes in ΔPost1M − Pre, especially DMN–ON, FPN–ON, SMN–SMN, and CON–ON (Figure 3K). Importantly, ΔPost1M − Post1W retained positive separation for FPN–FPN, FPN–SMN, and FPN–ON, suggesting that recovery was linked to continued beta‐band communication consolidation from 1 week to 1 month (Figure 3L).

Low‐frequency communication effects were weaker and less stable. Delta‐band ModularAUC showed mostly mild‐to‐moderate effects with several direction reversals across static time points and change windows, indicating poor temporal consistency (Figure S3A–F). Theta‐band effects were similarly scattered, with mixed positive and negative directions and no coherent cross‐network pattern (Figure S3G–L). Thus, unlike beta‐band communication architecture, delta/theta changes mainly reflected dispersed low‐frequency fluctuations rather than a core recovery‐related network pattern.

### 3.4 Correlation With Sustained Clinical Improvement

**FIGURE 4:**
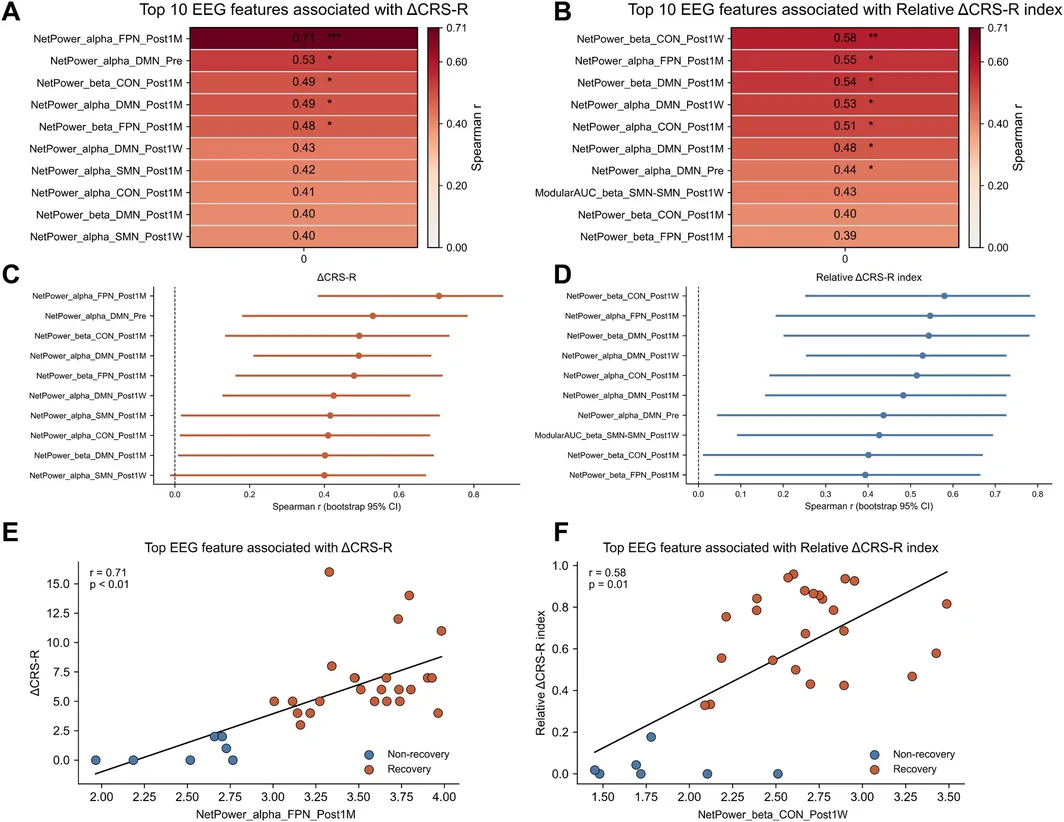
EEG features most strongly associated with long‐term clinical improvement. (A) The top 10 EEG features associated with ΔCRS‐R are dominated by high‐frequency NetPower features. (B) The top 10 EEG features associated with Relative ΔCRS‐R index are also mainly high‐frequency NetPower features, with limited contributions from communication features. (C) Bootstrap results confirm the robustness of the top EEG correlates of ΔCRS‐R. (D) Bootstrap results confirm the robustness of the top EEG correlates of Relative ΔCRS‐R index. (E) The strongest EEG correlate of ΔCRS‐R is NetPower_alpha_FPN_Post1M. (F) The strongest EEG correlate of Relative ΔCRS‐R index is NetPower_beta_CON_Post1W. ΔCRS‐R indicates CRS‐R total at 6 months minus CRS‐R total at baseline. Relative ΔCRS‐R index (%) indicates 100 × (CRS‐R index at 6 months − CRS‐R index at baseline)/CRS‐R index at baseline. NetPower denotes network‐level spectral power after log transformation. ModularAUC denotes threshold‐robust module‐interaction area under the curve. CI, confidence interval; CON, cingulo‐opercular network; CRS‐R, Coma Recovery Scale–Revised; DMN, default mode network; FPN, frontoparietal network; Post1M, 1 month after stimulation onset; Post1W, 1 week after stimulation onset; Pre, pre‐implantation baseline; *r*, Spearman correlation coefficient; SMN, somatomotor network. *p < 0.05, **p < 0.01, ***p < 0.001. Stars in panels (A) and (B) indicate significance after Benjamini–Hochberg false discovery rate correction.

For ΔCRS‐R total, all top 10 correlates were NetPower measures, led by NetPower_alpha_FPN_Post1M (*r* = 0.71, FDR‐significant), followed by NetPower_alpha_DMN_Pre (*r* = 0.53), NetPower_beta_CON_Post1M (*r* = 0.49), NetPower_alpha_DMN_Post1M (*r* = 0.49), and NetPower_beta_FPN_Post1M (*r* = 0.48), indicating an alpha‐dominant, beta‐supported energy signature of absolute clinical improvement (Figure 4A). For Relative ΔCRS‐R index, the strongest correlate was NetPower_beta_CON_Post1W (*r* = 0.58, FDR‐significant), followed by NetPower_alpha_FPN_Post1M (*r* = 0.55), NetPower_beta_DMN_Post1M (*r* = 0.54), NetPower_alpha_DMN_Post1W (*r* = 0.53), NetPower_alpha_CON_Post1M (*r* = 0.51), and NetPower_alpha_DMN_Post1M (*r* = 0.48); only one ModularAUC feature, ModularAUC_beta_SMN–SMN_Post1W, entered the top 10, suggesting a limited additional contribution of communication architecture (Figure 4B).

Robustness analyses supported these associations. Bootstrap confidence intervals for the top ΔCRS‐R total features remained positive, with NetPower_alpha_FPN_Post1M showing the strongest right‐shifted distribution (Figure 4C). For Relative ΔCRS‐R index, NetPower_beta_CON_Post1W, NetPower_alpha_FPN_Post1M, and NetPower_beta_DMN_Post1M showed similarly stable positive intervals (Figure 4D). Scatterplots confirmed the strongest covariate‐adjusted associations: NetPower_alpha_FPN_Post1M correlated with ΔCRS‐R total (*r* = 0.71, *p* < 0.01) (Figure 4E), and NetPower_beta_CON_Post1W correlated with Relative ΔCRS‐R index (*r* = 0.58, *p* = 0.01) (Figure 4F).

**FIGURE 5:**
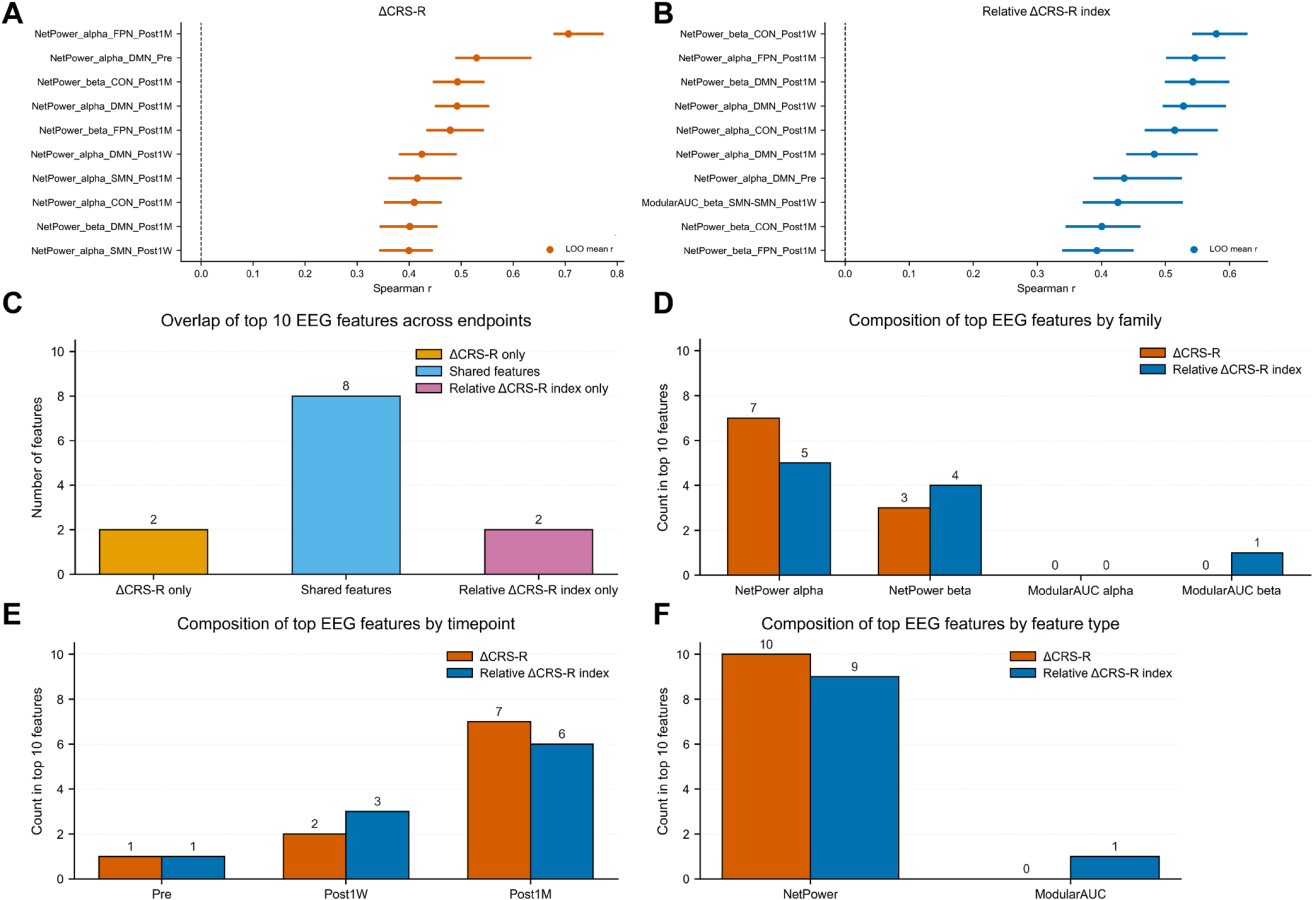
Robustness and composition of EEG features associated with long‐term clinical improvement. (A) Leave‐one‐out analysis shows that the top EEG features associated with ΔCRS‐R are stable across individual exclusions. (B) Leave‐one‐out analysis shows similar stability for the top EEG features associated with Relative ΔCRS‐R index. (C) The two clinical endpoints share a large proportion of top‐ranked EEG features. (D) Most top EEG features belong to the NetPower alpha and NetPower beta families. (E) Most top EEG features are concentrated at Post1M, with fewer features at Pre and Post1W. (F) NetPower contributes the majority of top‐ranked EEG features, whereas ModularAUC contributes only minimally. ΔCRS‐R indicates CRS‐R total at 6 months minus CRS‐R total at baseline. Relative ΔCRS‐R index (%) indicates 100 × (CRS‐R index at 6 months − CRS‐R index at baseline)/CRS‐R index at baseline. NetPower denotes network‐level spectral power after log transformation. ModularAUC denotes threshold‐robust module‐interaction area under the curve. LOO, leave‐one‐out; Post1M, 1 month after stimulation onset; Post1W, 1 week after stimulation onset; Pre, pre‐implantation baseline; *r*, Spearman correlation coefficient.

Leave‐one‐out analyses showed that these findings were not driven by single subjects: NetPower_alpha_FPN_Post1M and NetPower_beta_CON_Post1W retained the highest mean correlations for ΔCRS‐R total and Relative ΔCRS‐R index, respectively (Figure 5A,B). The two endpoints shared 8 of their top 10 EEG features (Figure 5C). Feature composition showed 7 NetPower‐alpha and 3 NetPower‐beta features for ΔCRS‐R total, versus 5 NetPower‐alpha, 4 NetPower‐beta, and 1 ModularAUC‐beta feature for Relative ΔCRS‐R index (Figure 5D). Top features were mainly post‐stimulation, especially Post1M: 1 Pre, 2 Post1W, and 7 Post1M features for ΔCRS‐R total, and 1 Pre, 3 Post1W, and 6 Post1M features for Relative ΔCRS‐R index (Figure 5E). Feature‐type analysis confirmed NetPower dominance, accounting for 10/10 and 9/10 top features for the two endpoints, respectively (Figure 5F). Thus, long‐term improvement was primarily coupled to alpha/beta network‐energy enhancement rather than broad communication‐architecture dominance. Exploratory etiology‐stratified analyses of the top‐ranked EEG features showed broadly consistent positive associations within the traumatic brain injury and stroke subgroups. The hypoxic injury subgroup was interpreted descriptively because of its small sample size (*n* = 4). None of the feature‐by‐etiology interaction effects survived Benjamini–Hochberg FDR correction (Figures S4 and S5).

## 4 Discussion

Using source‐space EEG, we established a NetPower–ModularAUC dual‐axis framework to characterize early brain‐network reorganization after SCS in patients with chronic disorders of consciousness and to relate these changes to 6‐month clinical outcomes. The results indicate that recovery‐related reorganization was primarily characterized by enhanced alpha/beta network energy and consolidated beta‐band communication architecture, with the strongest effects emerging at 1 month after stimulation. These recovery‐related features were mainly distributed across the FPN, DMN, and CON, suggesting that long‐term recovery after SCS depends more on sustained reconstruction of high‐frequency network function than on broad, nonspecific spectral changes.

Across feature types, frequency bands, and time points, the FPN, DMN, and CON emerged as the most consistent contributors to recovery‐related effects. The FPN was mainly implicated by alpha‐band NetPower and its association with long‐term clinical improvement, the DMN by broad alpha/beta energy enhancement and beta‐band cross‐network interactions, and the CON by early beta‐band NetPower related to relative clinical improvement. SMN and ON contributions were more evident in beta‐band communication architecture, particularly at Post1M, whereas delta/theta findings were less consistent across analyses.

In DoC, increased low‐frequency activity is commonly interpreted as a marker of severe brain dysfunction, impaired thalamocortical integration, or insufficient cortical activation [39, 40]. By contrast, alpha and beta activity are more closely associated with cortical excitability, large‐scale network integration, cognitive control, and efficient information transfer [41, 42, 43]. In the present study, the advantage observed in the Recovery group was not a broadband enhancement across all frequencies, but was selectively concentrated in alpha/beta activity. Delta/theta changes were less stable and more likely reflected diffuse brain injury, cortical inhibition, or slow‐wave burden [44]. Thus, effective network reorganization after SCS may represent a transition from a low‐frequency‐dominant, poorly integrated state toward a high‐frequency‐supported, more integrated state. Notably, beta‐band activity showed stronger discriminative value across multiple analytical levels, suggesting that it may more directly index the restoration of network integration and information exchange. In this sense, long‐term recovery is not simply explained by reduced slow‐wave activity, but by the re‐establishment and stabilization of high‐frequency network activity.

Physiologically, the predominance of alpha/beta NetPower may reflect a recovery‐relevant shift toward restored thalamocortical drive and cortical activation [44]. In DoC, excessive delta/theta activity is commonly associated with impaired thalamocortical integration and reduced cortical responsiveness, whereas preserved alpha/beta activity has been linked to more organized cortical dynamics and better clinical outcomes [25]. Therefore, the sustained alpha/beta NetPower increase observed after SCS may indicate strengthening of cortical excitability and large‐scale integrative capacity rather than a nonspecific broadband power increase.

On the energy‐state axis, the Recovery group showed higher alpha/beta NetPower across all five canonical networks, and this advantage further increased from 1 week to 1 month after stimulation, reaching its maximum at Post1M. This finding suggests that the critical distinction between Recovery and Non‐recovery patients is not merely whether the brain generates an early response to SCS, but whether this response can be consolidated into a stable high‐frequency network energy state. Mechanistically, SCS may not directly “produce” consciousness; rather, it may increase the background activation level of distributed brain networks and enrich alpha/beta oscillatory resources, thereby providing an energetic substrate for subsequent network integration and behavioral recovery. This high‐frequency enhancement was particularly stable in higher‐order networks, including the FPN, DMN, and CON. The FPN is involved in external task control and executive regulation, the DMN supports internal‐state maintenance and large‐scale integration, and the CON is closely related to arousal maintenance, salience detection, and state switching [45, 46, 47]. Sustained alpha/beta NetPower elevation in these networks therefore suggests that the recovered brain was not merely transiently “aroused”, but was progressively rebuilding a functional platform for cross‐network coordination.

Of note, beta‐band effects were strongest in the DMN, CON, and ON, indicating that recovery‐related high‐frequency enhancement was not confined to the frontoparietal control system, but also extended to posterior sensory‐integration networks. The involvement of the ON suggests that posterior cortical regions may represent important network nodes for consciousness recovery and behavioral expression [48]. In contrast, theta/delta NetPower generally decreased over time, while the Non‐recovery group often retained higher low‐frequency power, indicating that low‐frequency changes may reflect brain‐state dysregulation or slow‐wave burden rather than core drivers of recovery. The clinical relevance of the energy axis therefore lies in determining whether alpha/beta activity can be sustained and stabilized by 1 month after stimulation.

At the communication‐architecture level, the beta band provided the most informative recovery‐related pattern. Unlike the more localized and heterogeneous alpha‐band changes, beta‐band ModularAUC showed broader and more coherent enhancement of within‐ and between‐network module interactions at Post1M, particularly involving the DMN, FPN, SMN, and their coupling with the ON. This finding is important because consciousness recovery cannot depend solely on increased power within individual networks; it also requires the re‐establishment of inter‐network communication, selective coupling, and hierarchical coordination. Neurophysiologically, beta oscillations are frequently linked to long‐range coupling, anterior–posterior cortical coordination, maintenance‐related information transfer, and stable cognitive states [43, 49]. In this study, the Recovery group showed not only strengthened within‐network interactions, such as FPN–FPN and SMN–SMN, but also enhanced cross‐network interactions, including FPN–SMN, FPN–ON, DMN–ON, and CON–ON. These findings indicate that recovery depends on the reconstruction of selective large‐scale network synergy rather than isolated strengthening of single networks. The broad enhancement of FPN‐related interactions suggests that the executive‐control system may play an organizing role in network reconfiguration, whereas the involvement of ON‐related interactions indicates that posterior cortical and sensory‐integration systems may serve as important output pathways through which network reorganization supports behavioral improvement.

Change‐window analyses further showed that the core beta‐band differences were concentrated between 1 week and 1 month after stimulation, rather than in the earliest post‐stimulation phase. This suggests that long‐term recovery may not be determined by the initial neural response itself, but by whether that response can be progressively strengthened, integrated, and stabilized into a beta‐band communication advantage. By comparison, although delta/theta bands showed some localized differences, they lacked the broad, coherent, and temporally increasing structural pattern observed in the beta band. These findings support the view that recovery‐related reorganization is not broadband reconnection, but is preferentially expressed as the restoration of long‐range coordination and network organization supported by high‐frequency, particularly beta‐band, oscillations.

Beyond group comparisons, analyses of continuous clinical outcomes further confirmed the clinical relevance of high‐frequency EEG features. The features most strongly associated with ΔCRS‐R total and Relative ΔCRS‐R index were almost exclusively alpha/beta NetPower rather than ModularAUC, suggesting that individual differences in long‐term behavioral improvement are most directly coupled to a small set of high‐frequency network energy measures, particularly within the FPN, DMN, and CON. Specifically, NetPower_alpha_FPN_Post1M showed the strongest association with ΔCRS‐R total, potentially reflecting a stabilized network energy state after mid‐term consolidation. In contrast, NetPower_beta_CON_Post1W showed the strongest association with Relative ΔCRS‐R index, potentially indexing the early “launch capacity” of the salience/arousal regulatory system in response to SCS. Thus, FPN alpha enhancement may represent a consolidated recovery phenotype, whereas CON beta enhancement may serve as an early recovery‐sensitive marker. Moreover, 8 of the top 10 EEG features overlapped between the two clinical endpoints, indicating that this high‐frequency energy pattern is not dependent on a single outcome definition but shows cross‐endpoint consistency. This supports both the mechanistic interpretability and potential translational value of the dual‐axis framework.

Several high‐frequency features also showed visible baseline separation, suggesting that pre‐existing brain‐state organization may contribute to later recovery. Although these baseline differences did not reach statistical significance in the present cohort, they may reflect preserved neural reserve or a favorable baseline network configuration. In parallel, the longitudinal strengthening from Post1W to Post1M indicates that recovery may also depend on sustained post‐SCS network consolidation. We therefore interpret favorable outcome as the combined result of baseline network capacity and subsequent stimulation‐associated reorganization, a distinction that should be tested in larger cohorts and controlled designs.

The mechanisms by which SCS promotes recovery in DoC remain incompletely understood, but our findings suggest that its effect is unlikely to be a single‐pathway immediate response and may instead involve gradual reshaping of brain‐network states. Mechanistically, SCS may first enhance global arousal through spinal–brainstem–thalamocortical ascending systems, increasing the baseline excitability of large‐scale networks [50]. If this effect is transformed into sustained alpha/beta high‐frequency energy elevation, it may provide the basis for rebuilding inter‐network coordination [16]. Ultimately, long‐term behavioral improvement may become more likely when this high‐frequency advantage further consolidates into stable beta‐band communication architecture [51]. Thus, source‐space EEG, particularly alpha/beta NetPower and beta ModularAUC between 1 week and 1 month after stimulation, may identify effective network reorganization earlier than CRS‐R assessment alone and support a dual‐dimensional behavior–EEG network framework for evaluating therapeutic response. However, the sample size was modest, the Non‐recovery group was small, and etiological heterogeneity was substantial. The small Non‐recovery group limited statistical power and increased the uncertainty of between‐group effect estimates. Therefore, the ModularAUC findings should be interpreted as exploratory network‐level evidence rather than definitive recovery biomarkers. EEG was recorded with stimulation turned off and therefore reflects an offline brain state rather than an immediate online response. This framework requires validation in larger, more balanced external cohorts.

In conclusion, SCS‐related brain‐network reorganization in DoC is not a nonspecific broadband effect, but is marked by alpha/beta energy enhancement and beta‐band communication consolidation. Favorable recovery was associated with sustained high‐frequency network activity, broader beta‐band synergy, and stronger EEG–behavior coupling, supporting the dual‐axis source‐space EEG framework as a mechanistically informative tool for monitoring SCS‐related recovery.

## Author Contributions

Kang Zhang and Li Bie conceived and designed the study. Zean Li and Xinran Zhang wrote the manuscript, which was revised by Li Bie and approved by all the authors. Zhiyang Xu and Chunyun Zhang contributed to data collection. Chuanxiang Lv, Boyuan Zhang, Yunqian Li, and Haoyong Jin performed statistical analyses of the data.

## Funding

This work was supported by Science and Technology Development Plan Project of Jilin Province, China under Grant (20230204094YY) and (YDZJ202201ZYTS001).

## Ethics Statement

The study was approved by the Ethics Committee of the First Hospital of Jilin University (KYSQ 2021‐396‐01) and was conducted in accordance with the Declaration of Helsinki.

## Consent

Written informed consent was obtained from the legally authorized representatives of all participants.

## Conflicts of Interest

The authors declare no conflicts of interest.

## Supporting information


**Figure S1:** Brain template used for source reconstruction and network mapping. Regions of interest from the Dosenbach atlas are assigned to the five canonical functional networks, including the default mode, frontoparietal, cingulo‐opercular, somatomotor, and occipital networks. CON, cingulo‐opercular network; DMN, default mode network; FPN, frontoparietal network; ON, occipital network; SMN, somatomotor network.

**Figure S2:** Low‐frequency energy‐state changes in the theta and delta bands. (A) Theta‐band NetPower shows an overall decline over time in both groups. (B) Delta‐band NetPower shows a similar declining pattern. (C) Effect‐size summary of theta‐band NetPower indicates predominantly negative and weak between‐group differences. (D) Effect‐size summary of delta‐band NetPower also shows predominantly negative and inconsistent between‐group differences. NetPower denotes network‐level spectral power after log transformation. CON, cingulo‐opercular network; DMN, default mode network; FPN, frontoparietal network; ON, occipital network; Post1M, 1 month after stimulation onset; Post1W, 1 week after stimulation onset; Pre, pre‐implantation baseline; s.e.m., standard error of the mean; SMN, somatomotor network. Cohen's *d* indicates between‐group effect size (Recovery − Non‐recovery).

**Figure S3:** Low‐frequency communication‐architecture changes in the delta and theta bands. (A–F) Delta‐band module‐interaction AUC shows weak and spatially inconsistent between‐group differences across static time points and change windows. (G–L) Theta‐band module‐interaction AUC also shows heterogeneous and unstable patterns, without the broad recovery‐related enhancement observed in the beta band. AUC, area under the threshold curve; CON, cingulo‐opercular network; DMN, default mode network; FPN, frontoparietal network; ON, occipital network; Post1M, 1 month after stimulation onset; Post1W, 1 week after stimulation onset; Pre, pre‐implantation baseline; SMN, somatomotor network. Δ indicates change between two time points. Hedges' *g* indicates between‐group effect size (Recovery − Non‐recovery).

**Figure S4:** Etiology‐stratified correlations between top‐ranked EEG features and long‐term clinical improvement. (A) Etiology‐stratified Spearman correlations between the top 10 Spearman‐ranked EEG features and ΔCRS‐R total. Points indicate within‐etiology Spearman correlation coefficients, and horizontal lines indicate bootstrap 95% confidence intervals. (B) Etiology‐stratified Spearman correlations between the top 10 Spearman‐ranked EEG features and the Relative ΔCRS‐R index. (C) Heatmap showing within‐etiology Spearman r values for the top 10 EEG features associated with ΔCRS‐R total. (D) Heatmap showing within‐etiology Spearman r values for the top 10 EEG features associated with the Relative ΔCRS‐R index. Colors denote injury etiology: TBI, stroke, and hypoxic injury. Because the hypoxic injury subgroup included only four patients, its estimates were considered descriptive only. Asterisks indicate uncorrected within‐stratum significance levels: *p < 0.05, **p < 0.01, ***p < 0.001. ΔCRS‐R indicates CRS‐R total at 6 months minus CRS‐R total at baseline.

**Figure S5:** Etiology‐stratified scatter plots for the leading EEG features associated with clinical improvement. (A) Scatter plots showing the relationship between NetPower_alpha_FPN_Post1M and ΔCRS‐R total within traumatic brain injury, stroke, and hypoxic injury subgroups. (B) Scatter plots showing the relationship between NetPower_beta_CON_Post1W and the Relative ΔCRS‐R index within each etiological subgroup. Points indicate individual patients. Colors denote injury etiology, and marker shapes denote clinical outcome group: circles indicate Recovery and squares indicate Non‐recovery. Black lines indicate linear trend lines within each etiological subgroup. Spearman *r* and *p* values are shown within each panel. Because the hypoxic injury subgroup included only four patients, its correlation estimates were interpreted descriptively. CRS‐R, Coma Recovery Scale–Revised; TBI, traumatic brain injury.

## Data Availability

The data that support the findings of this study are available on request from the corresponding author. The data are not publicly available due to privacy or ethical restrictions.

## References

[1] J. T. Giacino , J. J. Fins , S. Laureys , and N. D. Schiff , “Disorders of Consciousness After Acquired Brain Injury: The State of the Science,” Nature Reviews. Neurology 10, no. 2 (2014): 99–114, 10.1038/nrneurol.2013.279. 24468878

[2] S. Septien and M. A. Rubin , “Disorders of Consciousness: Ethical Issues of Diagnosis, Treatment, and Prognostication,” Seminars in Neurology 38, no. 5 (2018): 548–554, 10.1055/s-0038-1667384. 30321893

[3] J. L. Bernat , “Chronic Disorders of Consciousness,” Lancet 367, no. 9517 (2006): 1181–1192, 10.1016/S0140-6736(06)68508-5. 16616561

[4] S. Laureys , G. G. Celesia , F. Cohadon , et al., “Unresponsive Wakefulness Syndrome: A New Name for the Vegetative State or Apallic Syndrome,” BMC Medicine 8 (2010): 68, 10.1186/1741-7015-8-68. 21040571 PMC2987895

[5] W. J. Burke , “The Minimally Conscious State: Definition and Diagnostic Criteria,” Neurology 59, no. 9 (2002): 1473. 12427915

[6] J. T. Giacino , K. Kalmar , and J. Whyte , “The JFK Coma Recovery Scale‐Revised: Measurement Characteristics and Diagnostic utility11No Commercial Party Having a Direct Financial Interest in the Results of the Research Supporting This Article Has or Will Confer a Benefit Upon the Authors or Upon Any Organization With Which the Authors Are Associated,” Archives of Physical Medicine and Rehabilitation 85, no. 12 (2004): 2020–2029, 10.1016/j.apmr.2004.02.033. 15605342

[7] Y. Zhang , J. Wang , C. Schnakers , et al., “Validation of the Chinese Version of the Coma Recovery Scale‐Revised (CRS‐R),” Brain Injury 33, no. 4 (2019): 529–533, 10.1080/02699052.2019.1566832. 30663434

[8] A. Thibaut , N. Schiff , J. Giacino , S. Laureys , and O. Gosseries , “Therapeutic Interventions in Patients With Prolonged Disorders of Consciousness,” Lancet Neurology 18, no. 6 (2019): 600–614, 10.1016/S1474-4422(19)30031-6. 31003899

[9] A. Thibaut , M. Bruno , D. Ledoux , A. Demertzi , and S. Laureys , “tDCS in Patients With Disorders of Consciousness: Sham‐Controlled Randomized Double‐Blind Study,” Neurology 82, no. 13 (2014): 1112–1118, 10.1212/WNL.0000000000000260. 24574549

[10] A. Naro , P. Bramanti , A. Leo , M. Russo , and R. S. Calabro , “Transcranial Alternating Current Stimulation in Patients With Chronic Disorder of Consciousness: A Possible Way to Cut the Diagnostic Gordian Knot?,” Brain Topography 29, no. 4 (2016), 10.1007/s10548-016-0489-z. 27062669

[11] T. Kanno , I. Morita , S. Yamaguchi , et al., “Dorsal Column Stimulation in Persistent Vegetative State,” Neuromodulation: Technology at the Neural Interface 12, no. 1 (2009): 33–38, 10.1111/j.1525-1403.2009.00185.x. 22151220

[12] Y. Xu , P. Li , S. Zhang , et al., “Cervical Spinal Cord Stimulation for the Vegetative State: A Preliminary Result of 12 Cases,” Neuromodulation: Technology at the Neural Interface 22, no. 3 (2019): 347–354, 10.1111/ner.12903. 30548939

[13] K. Funahashi , N. Komai , M. Ogura , T. Kuwata , M. Nakai , and N. Tsuji , “Effects and Indications of Spinal Cord Stimulation on the Vegetative Syndrome,” No Shinkei Geka 17, no. 10 (1989): 917–923. 2812256

[14] Y. Hosobuchi , “Electrical Stimulation of the Cervical Spinal Cord Increases Cerebral Blood Flow in Humans,” Applied Neurophysiology 48, no. 1–6 (1985): 372–376, 10.1159/000101161. 3879799

[15] M. Visocchi , T. Tartaglione , R. Romani , and M. Meglio , “Spinal Cord Stimulation Prevents the Effects of Combined Experimental Ischemic and Traumatic Brain Injury. An MR Study,” Stereotactic and Functional Neurosurgery 76, no. 3–4 (2001): 276–281, 10.1159/000066731. 12378109

[16] Y. Bai , X. Xia , Z. Liang , et al., “Frontal Connectivity in EEG Gamma (30–45 Hz) Respond to Spinal Cord Stimulation in Minimally Conscious State Patients,” Frontiers in Cellular Neuroscience 11 (2017): 177, 10.3389/fncel.2017.00177. 28701924 PMC5487422

[17] T. Yamamoto , Y. Katayama , T. Obuchi , K. Kobayashi , H. Oshima , and C. Fukaya , “Spinal Cord Stimulation for Treatment of Patients in the Minimally Conscious State,” Neurologia Medico‐Chirurgica 52, no. 7 (2012): 475–481, 10.2176/nmc.52.475. 22850495

[18] B. Guerra‐Carrillo , A. P. Mackey , and S. A. Bunge , “Resting‐State fMRI: A Window Into Human Brain Plasticity,” Neuroscientist 20, no. 5 (2014): 522–533, 10.1177/1073858414524442. 24561514

[19] X. Duan , S. He , W. Liao , et al., “Reduced Caudate Volume and Enhanced Striatal‐DMN Integration in Chess Experts,” NeuroImage 60, no. 2 (2012): 1280–1286, 10.1016/j.neuroimage.2012.01.047. 22270350

[20] M. Taubert , G. Lohmann , D. S. Margulies , A. Villringer , and P. Ragert , “Long‐Term Effects of Motor Training on Resting‐State Networks and Underlying Brain Structure,” NeuroImage 57, no. 4 (2011): 1492–1498, 10.1016/j.neuroimage.2011.05.078. 21672633

[21] C. Luo , Z. W. Guo , Y. X. Lai , et al., “Musical Training Induces Functional Plasticity in Perceptual and Motor Networks: Insights From Resting‐State FMRI,” PLoS One 7, no. 5 (2012), 10.1371/journal.pone.0036568. PMC3346725 22586478

[22] O. Sporns , “Network Attributes for Segregation and Integration in the Human Brain,” Current Opinion in Neurobiology 23, no. 2 (2013): 162–171, 10.1016/j.conb.2012.11.015. 23294553

[23] “Communities, Modules and Large‐Scale Structure in Networks,” *Nature Physics*, April 26, 2026, https://www.nature.com/articles/nphys2162.

[24] M. Veciana de Las Heras , J. Sala‐Padro , J. Pedro‐Perez , B. García‐Parra , G. Hernández‐Pérez , and M. Falip , “Utility of Quantitative EEG in Neurological Emergencies and ICU Clinical Practice,” Brain Sciences 14, no. 9 (2024): 939, 10.3390/brainsci14090939. 39335433 PMC11430096

[25] S. Chennu , J. Annen , S. Wannez , et al., “Brain Networks Predict Metabolism, Diagnosis and Prognosis at the Bedside in Disorders of Consciousness,” Brain: A Journal of Neurology 140, no. 8 (2017): 2120–2132, 10.1093/brain/awx163. 28666351

[26] B. Díaz‐Fernández , D. Henao‐Herreno , J. Nieto , et al., “Differentiation of Tumor Versus Peritumoral Cortex in Gliomas by Intraoperative Electrocorticography,” Neuro‐Oncology 27, no. 7 (2025): 1758–1771, 10.1093/neuonc/noaf082. 40127031 PMC12417838

[27] S. M. Gorka , J. Jimmy , K. Koning , et al., “Alterations in Large‐Scale Resting‐State Network Nodes Following Transcranial Focused Ultrasound of Deep Brain Structures,” Frontiers in Human Neuroscience 18 (2024), 10.3389/fnhum.2024.1486770. PMC11652661 39698148

[28] A. E. Warren , M. Raguž , H. Friedrich , et al., “A Human Brain Network Linked to Restoration of Consciousness After Deep Brain Stimulation,” Nature Communications 16, no. 1 (2025), 10.1038/s41467-025-61988-4. PMC12279989 40691170

[29] W. J. Neumann , A. Horn , and A. A. Kühn , “Insights and Opportunities for Deep Brain Stimulation as a Brain Circuit Intervention,” Trends in Neurosciences 46, no. 6 (2023): 472–487, 10.1016/j.tins.2023.03.009. 37105806

[30] X. Wu , H. Duan , Y. Wang , S. She , Y. Hu , and B. He , “Default Mode Network and Visual Network Responsiveness to Transcutaneous Auricular Vagus Nerve Stimulation Predict Its Variable Efficacy in Primary Insomnia Disorder,” Frontiers in Neurology 16 (2025), 10.3389/fneur.2025.1703747. PMC12747963 41476747

[31] B. Wutzl , S. M. Golaszewski , K. Leibnitz , et al., “Narrative Review: Quantitative EEG in Disorders of Consciousness,” Brain Sciences 11, no. 6 (2021), 10.3390/brainsci11060697. PMC8228474 34070647

[32] D. Szirmai , A. Zabihi , T. Kói , et al., “EEG Connectivity and Network Analyses Predict Outcome in Patients With Disorders of Consciousness—A Systematic Review and Meta‐Analysis,” Heliyon 10, no. 10 (2024), 10.1016/j.heliyon.2024.e31277. PMC11141356 38826755

[33] H. Li , L. Dong , W. Su , et al., “Multiple Patterns of EEG Parameters and Their Role in the Prediction of Patients With Prolonged Disorders of Consciousness,” Frontiers in Neuroscience 19 (2025): 1492225, 10.3389/fnins.2025.1492225. 39975972 PMC11836006

[34] J. Annen , M. M. Filippini , E. Bonin , et al., “Diagnostic Accuracy of the CRS‐R Index in Patients With Disorders of Consciousness,” Brain Injury 33, no. 11 (2019): 1409–1412, 10.1080/02699052.2019.1644376. 31319707

[35] A. Delorme and S. Makeig , “EEGLAB: An Open Source Toolbox for Analysis of Single‐Trial EEG Dynamics Including Independent Component Analysis,” Journal of Neuroscience Methods 134, no. 1 (2004): 9–21, 10.1016/j.jneumeth.2003.10.009. 15102499

[36] R. Oostenveld , P. Fries , E. Maris , and J. M. Schoffelen , “FieldTrip: Open Source Software for Advanced Analysis of MEG, EEG, and Invasive Electrophysiological Data,” Computational Intelligence and Neuroscience 2011 (2011), 10.1155/2011/156869. PMC3021840 21253357

[37] N. U. F. Dosenbach , B. Nardos , A. L. Cohen , et al., “Prediction of Individual Brain Maturity Using fMRI,” Science 329, no. 5997 (2010): 1358–1361, 10.1126/science.1194144. 20829489 PMC3135376

[38] “GRETNA: A Graph Theoretical Network Analysis Toolbox for Imaging Connectomics,” April 26, 2026, https://pubmed.ncbi.nlm.nih.gov/26175682/. 10.3389/fnhum.2015.00386 PMC4485071 26175682

[39] J. Frohlich , D. Toker , and M. M. Monti , “Consciousness Among Delta Waves: A Paradox?,” Brain: A Journal of Neurology 144, no. 8 (2021), 10.1093/brain/awab095. 33693596

[40] A. Duszyk‐Bogorodzka , M. Zieleniewska , and K. Jankowiak‐Siuda , “Brain Activity Characteristics of Patients With Disorders of Consciousness in the EEG Resting State Paradigm: A Review,” Frontiers in Systems Neuroscience 16 (2022), 10.3389/fnsys.2022.654541. PMC9198636 35720438

[41] J. I. Chapeton , R. Haque , J. H. Wittig , S. K. Inati , and K. A. Zaghloul , “Large‐Scale Communication in the Human Brain Is Rhythmically Modulated Through Alpha Coherence,” Current Biology: CB 29, no. 17 (2019): 2801–2811, 10.1016/j.cub.2019.07.014. 31422882 PMC6736747

[42] S. Sadaghiani and A. Kleinschmidt , “Brain Networks and α‐Oscillations: Structural and Functional Foundations of Cognitive Control,” Trends in Cognitive Sciences 20, no. 11 (2016): 805–817, 10.1016/j.tics.2016.09.004. 27707588

[43] A. K. Engel and P. Fries , “Beta‐Band Oscillations—Signalling the Status Quo?,” Current Opinion in Neurobiology 20, no. 2 (2010): 156–165, 10.1016/j.conb.2010.02.015. 20359884

[44] N. D. Schiff , “Recovery of Consciousness After Brain Injury: A Mesocircuit Hypothesis,” Trends in Neurosciences 33, no. 1 (2010): 1–9, 10.1016/j.tins.2009.11.002. 19954851 PMC2931585

[45] V. Menon , “Large‐Scale Brain Networks and Psychopathology: A Unifying Triple Network Model,” Trends in Cognitive Sciences 15, no. 10 (2011): 483–506, 10.1016/j.tics.2011.08.003. 21908230

[46] “The Cognitive Control Network: Integrated Cortical Regions With Dissociable Functions,” April 26, 2026, https://pubmed.ncbi.nlm.nih.gov/17553704/. 10.1016/j.neuroimage.2007.03.071 17553704

[47] D. A. Gusnard and M. E. Raichle , “Searching for a Baseline: Functional Imaging and the Resting Human Brain,” Nature Reviews Neuroscience 2, no. 10 (2001), 10.1038/35094500. 11584306

[48] L. Mencarelli , M. C. Biagi , R. Salvador , et al., “Network Mapping of Connectivity Alterations in Disorder of Consciousness: Towards Targeted Neuromodulation,” Journal of Clinical Medicine 9, no. 3 (2020): 828, 10.3390/jcm9030828. 32197485 PMC7141258

[49] A. Schnitzler and J. Gross , “Normal and Pathological Oscillatory Communication in the Brain,” Nature Reviews. Neuroscience 6, no. 4 (2005): 285–296, 10.1038/nrn1650. 15803160

[50] J. Si , Y. Dang , Y. Zhang , et al., “Spinal Cord Stimulation Frequency Influences the Hemodynamic Response in Patients With Disorders of Consciousness,” Neuroscience Bulletin 34, no. 4 (2018), 10.1007/s12264-018-0252-4. PMC6060214 29995275

[51] Y. Zhuang , Q. Ge , Q. Li , et al., “Combined Behavioral and EEG Evidence for the 70 Hz Frequency Selection of Short‐Term Spinal Cord Stimulation in Disorders of Consciousness,” CNS Neuroscience & Therapeutics 30, no. 2 (2024), 10.1111/cns.14388. PMC10848050 37563991

